# A self‐portrait: Design opportunities for a tool that supports children's involvement in brain‐related health care

**DOI:** 10.1111/hex.13431

**Published:** 2022-01-27

**Authors:** Paul Meulendijks, Neeltje E. M. van Haren, Mathieu A. Gielen, Marie‐Lise C. van Veelen‐Vincent

**Affiliations:** ^1^ Department of Industrial Design Engineering Delft University of Technology Delft The Netherlands; ^2^ Department of Child and Adolescent Psychiatry/Psychology Erasmus University Medical Center Rotterdam The Netherlands; ^3^ Department of Neurosurgery Erasmus University Medical Center Rotterdam The Netherlands; ^4^ Present address: Paul Meulendijks, MSc Philips Experience Design Eindhoven The Netherlands

**Keywords:** brain disorders, codesign, paediatric patients, patient participation, research through design, value‐based healthcare

## Abstract

**Introduction:**

Paediatric patients with disorders that involve brain functioning are particularly vulnerable with respect to including them in shared decision‐making. Current tools are mostly paper or digital patient information. We lay the groundwork for improving engagement with a concept that we coined ‘the Self‐Portrait’. The main goals were to identify (1) obstacles and (2) design parameters that enable patient participation.

**Methods:**

A research‐through‐design approach was utilized in nine patients with brain‐related disorders (4–12 years), 15 parents and 15 medical professionals, involving contextual research (interviews and observations) within the paediatric hospital and patients' homes and codesign. Sensitizing materials and early instances of design solutions were deployed as catalysts for communication. Five rounds of enriched interviews and design reviews were thematically analysed to answer the research questions.

**Results:**

Obstacles to child involvement were related to children's level of understanding, the time and energy necessary for information processing and lack of perceived relevance of the information. Patients' engagement is supported by design features that extend the time frame of interaction beyond the consultation, transfer information interactively and give control and influence during the consultation.

**Conclusion:**

Obstacles were detected that complicate child engagement, which differ between stakeholders. Promising design features were identified that have the potential to play an important role in enabling active child involvement. These findings show that applying principles of human‐centred design research and codesign can bring together patients, parents and medical professionals around a tool that provides a shared language and focus, which are prerequisites to increase child engagement.

**Patient or Public Contribution:**

Patients, parents and clinicians contributed as design informants during contextual research and design reviews. Clinicians provided feedback on the initial outcomes of thematic analysis. Two researchers assisted in consensus sessions during the thematic analysis.

## 1 INTRODUCTION

Shared decision‐making and value‐based healthcare are important practices that have been introduced in healthcare in the last decades.^1^ These practices aim to tailor medical care to the specific needs and characteristics of individual patients and their personal circumstances. Key to successfully implementing shared decision‐making is promoting active engagement of patients by sharing knowledge about their disease and the treatment options, but also to ask for personal preferences and values of the patient.^2^, ^3^ Currently, tools to engage patients are mostly paper or digital patient information and decision aids combined with counselling by a healthcare provider.^2^, ^3^

Shared decision‐making in paediatric healthcare comes with its own challenges, such as engaging both parents and children.^4^ Child participation is one of the five fundamental principles in the European guidelines on child healthcare.^5^ In the Netherlands, law regulations require healthcare providers to actively involve children in decision‐making from the age of 12, although younger children can profit from active participation as well.^6^ A review on shared decision‐making in a paediatric setting showed that most interventions are directed at clinicians and parents; only one in four interventions involved the child, mostly together with the parent.^1^, ^7^, ^8^ Children want to be involved and can provide valuable insights into how they experience their health and care.^6^, ^9^ Tapping into that source may improve treatment choices and treatment adherence.^10^

When and how to engage an ill child is challenging. Tools to transfer knowledge and understanding of disease and treatment must be adapted to the age and developmental level of the child.^11^ Careful evaluation of what is helpful to improve engagement and the decision‐making process, but not harmful, is necessary in each patient. Parents also play a role in engaging their child and face the challenge of balancing their knowledge and emotions against trusting their child's involvement and decision‐making.^1^, ^3^, ^12^

Tools have been developed to inform children, guide them through a care path and motivate and encourage them, such as booklets, brochures, videos or tokens.^6^, ^13^ These are mostly aimed at providing information and reducing anxiety and not specifically developed to encourage communication and interaction within the triad of the child, parents and professional.

The current project arose from the wish to involve paediatric patients in decisions that are based on the results of tests obtained in the *Child Brain Lab* (part of the Paediatric Brain Centre at Erasmus University Medical Centre, Sophia Children's Hospital). In the Child Brain Lab, brain function and development across several functional domains is tested in a playful circuit. The Pediatric Brain Center is a collaboration of multidisciplinary teams, taking care of a wide variety of brain‐related diagnoses and focussed on improving function and societal participation. Despite the diversity of disorders, patients share many of their functional and behavioural problems. These children are considered an especially vulnerable patient group with respect to patient involvement, as their medical condition causes additional challenges for them to understand their very condition (e.g., low developmental age). Cooperation with the Delft University of Technology was initiated for their capacity to design for people's well‐being, based on principles of human‐centred design research and codesign, also with children. We set out to design a tool to prepare 6–12‐year‐old children who visit the *Child Brain Lab* on the tests that they will undergo as well as to inform them on the results in an engaging and personally meaningful way. We aim to increase involvement and active participation during consultations, while also supporting counselling by the clinician. Here, we describe the preparatory groundwork for such a tool, which we coined the *‘Self‐Portrait*’.

The goals of the current project were (1) to identify obstacles for patient involvement and (2) to identify design parameters that enable patient participation. This is done using a research‐through‐design approach involving codesign,^14^ where contextual research and early instances of design solutions are deployed as catalysts for future users (patients, parents and medical professionals) to act as design informants^15^ and communicate their needs and desires.

## 2 MATERIALS AND METHODS

### 2.1 Design research set‐up

The project adopted an open, qualitative approach to disclose current obstacles (research question [RQ] 1) and future enablers of child participation (RQ2), viewed from the perspective of the triad of stakeholders: patients, their parents and medical professionals. Table 1 shows how the different research activities relate to the two overall research questions and how many stakeholders took part. All research activities were initiated and performed by P. M. We used a mix of design research techniques.^16^, ^17^, ^18^, ^19^ Stakeholders' current experiences are elicited through contextual research, primarily through observations of clinical practice combined with what we will call ‘enriched interviews’: interviews supported by generative techniques and materials,^16^ in part adapted to child participants.^17^

**Table 1:** An overview of the research questions, research activities, support material and number of stakeholders that were involved in each activity

Research questions	Research activity	Support materials	Participants		
			Children	Parents	Professionals
RQ1. What are the current obstacles of child involvement in patient consultations in the context of the Child Brain Lab?	A: Contextual observations		7	12	2
	B: Contextual interviews (child & parents: 35–90 min; professionals: 15–60 min)	Sensitizing booklet for children, brief survey for parents	6 (incl. 1 former child patient	8	8
RQ2. Which design features enable active child involvement?	C: 1st enriched online interviews (20–70 min)	Idea sketches of design features	1	5	5
	D1 2nd enriched online interviews (30–90 min)	Concept design sketches, highlighting concept features		9	7
	D2: 2nd enriched home interviews (50–90 min)	Physical prototype of full concept design (brain puzzle)	5	8	

### 2.2 Participants

Patients and their parents were recruited, based on age and willingness and ability to cooperate, through their clinicians. We aimed at a representation of all children and disciplines visiting the Child Brain Lab. Nine patients with brain‐related disorders (e.g., psychiatric, neurological or craniofacial; mean age: 7.3 years, range: 4–12 years), their parents (*N* = 15) and medical professionals (*N* = 15, with eight being physicians) participated in one or more research activities (see Tables S1 and S2).

### 2.3 Materials

Before the interviews, we gave out sensitizing materials^18^ (e.g., booklets for children, questions on daily life for parents, patient personas^19^ for clinicians). Sensitizing materials feature small playful and open‐ended assignments that help the participant to reflect on their experience regarding the object of the study. Early design sketches and models were used to concretize design features and proposed interactions. These designs were based on findings from initial field research and applied in consecutive rounds of research.

### 2.4 Research activities and support materials

*Observations* (A) of five consultation sessions during outpatient clinic visits and two electroencephalography (EEG) examinations focused on the interaction between the medical professionals, the parents and the child. Quick notes and sketches were made, which were refined afterwards (Figure S1).

*Contextual interviews* (B) with patients and parents took place in person. To structure the interview, children received a sensitizing booklet containing questions about their experiences and perspectives on child involvement in general and in their own care (Figure S2). Parents received a brief survey with questions on how they perceived the involvement of their child in general and in his or her own care.

**Figure 1:**
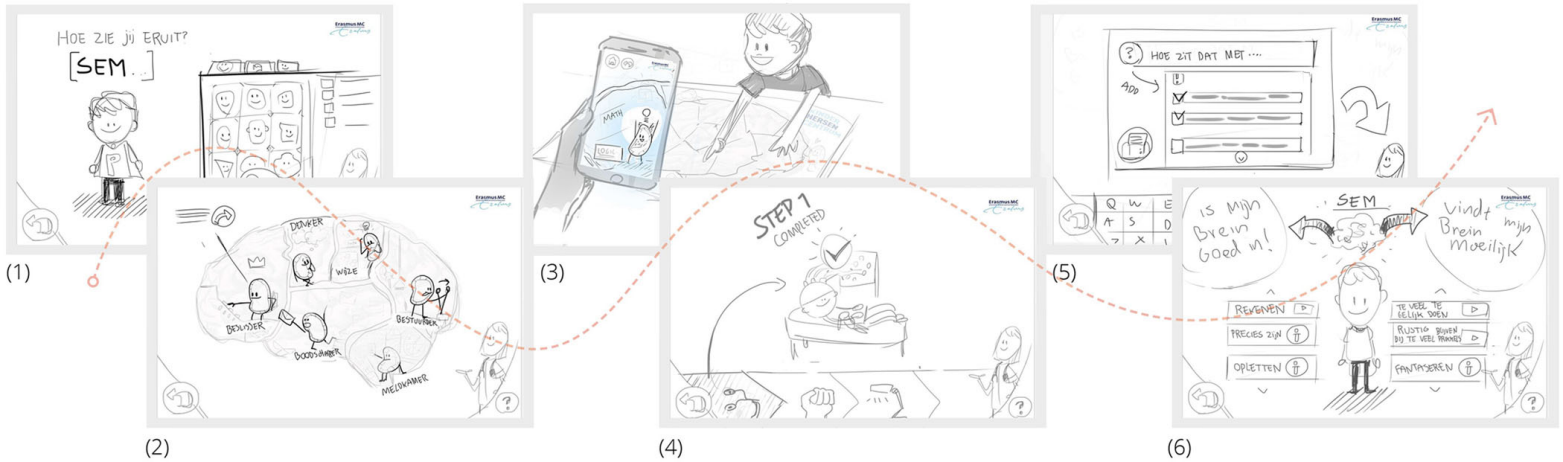
Impression of drawings of the design features. From left to right: (1) personal avatar to engage the child, (2) brain pictures to introduce the different brain functions, (3) interactive invite from the hospital to engage the child, (4) game in which the child can prepare for the test, (5) feature that stimulates the child to prepare questions that can be asked during the consultation and (6) overview explaining the child's strengths and weaknesses

*First enriched online interviews* (C) with patients, parents and clinicians were supported by drawings of different design features (Figure 1). These illustrated how a digital application in the home context could prepare a child for upcoming clinical tests as well as how the test results could be communicated. The design was aimed at connecting the different parts of the patient journey.

**Figure 2:**
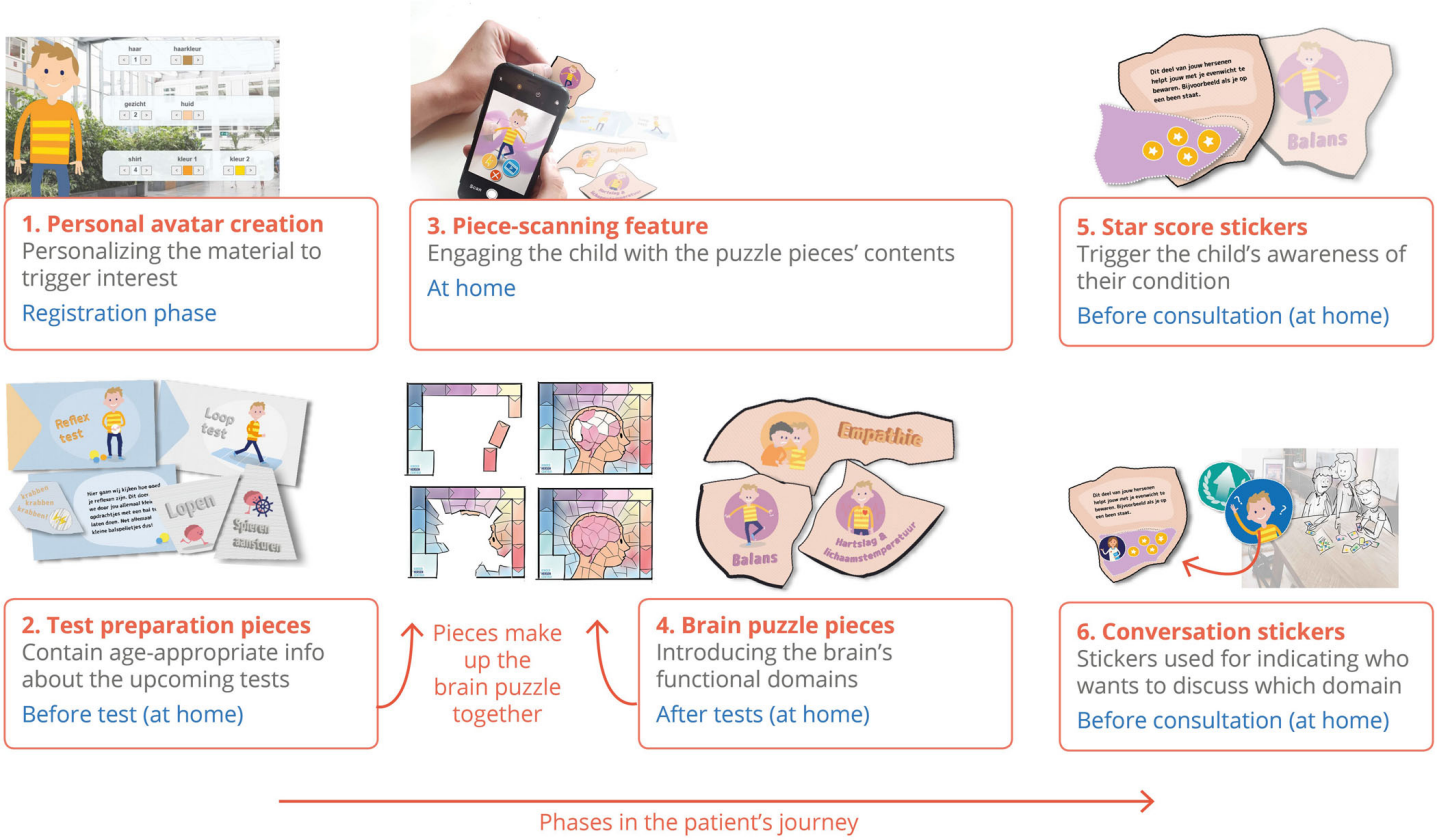
Overview of design features in the concept design organized by phase in the patient journey

*Second enriched online interviews* (D1) were supported by drawings of one coherent concept design: A brain puzzle that the child patients collect in pieces as they go through their clinical tests. The puzzle was designed to support each step of the patient journey: From preparing for tests at home, learning about brain functions and increasing awareness of the test outcomes to marking topics for discussion during the consultation (Figure 2). Participants were invited to write feedback on the design features before the interview (Figure S3).

The medical professionals received additional sensitizing material, which consisted of a concise textual overview of a fictitious patient's test results (Figure S4). They wrote down what they would discuss during a consultation with the fictitious patient and his family. Responses were used to concretize the interviews, focusing on the concept features that related most to them: The brain pieces, the star score stickers and the consultation (Figure S3). The interviews were unstructured; the content and duration differed depending on the professional's specialization and availability.

*Second enriched home interviews* (D2) introduced a physical prototype of the brain puzzle concept (Figure S5), which was shared and discussed with the child at their homes (except for one, which was held online). Puzzle pieces were personalized with an avatar of the child's own creation. Star scores on brain puzzle pieces reflected the child's performance on various tests. Conversation stickers could be attached to each brain puzzle piece to indicate a desire to discuss it with the clinician. Subsequently, parents were interviewed on their child's responses to the design features.

### 2.5 Analyses

The research was initially reported through the lens of an academic design‐research process as a master thesis.^20^ In writing this paper, the data were revisited, and documentation was restructured to allow analyses based on principles of thematic analysis.^21^ The collected data consisted of observation notes, written feedback on the design features, interviewer notes, audio recordings and in some cases video recordings of interviews. Partial or full transcripts were made. All texts were processed by selecting quotes relevant to the research focus, paraphrasing, labelling and clustering them into emerging topics and overarching themes, as is customary in generative design research.^16^ In the current project, we prioritized diverse and rich insights over saturation of each theme. Exhaustive development of the themes was deemed secondary to identifying opportunities related to the design challenge.

Responses from three of five sessions to the physical prototypes were interpreted by the main researcher (P. M.) and one of two independent researchers, based on a set of questions (e.g., ‘Did the child seem excited when he received his puzzle’; see Table S3A, B). The answers between two raters initially overlapped by 70%. The remaining 30% of questions were discussed in a consensus session, leading to final overlap in 97% of all questions.

### 2.6 Ethical considerations

Informed consent was given verbally by all participants for the contextual observations and the first enriched interview. For the interviews, parents signed a consent form and verbally agreed to audio recordings. The child assented verbally after the aim and procedure were explained to him or her in an age‐appropriate manner. Whenever a child seemed unwilling to continue the interview, the interview was terminated. The medical professionals gave verbal consent for audio recording the interview, after the context of the data collection was explained to them. Participants did not receive a participation fee (financial or otherwise) for their input.

This study was not subject to the Dutch law on medical research in humans (WMO) because participants were not exposed to procedures nor where they required to follow rules of behaviour (https://english.ccmo.nl/investigators/legal‐framework‐for‐medical‐scientific‐research/your‐research‐is‐it‐subject‐to‐the‐wmo‐or‐not).

## 3 RESULTS

The dataset contained 26 h of audio recordings plus observation notes from semi‐structured interviews. From six h also video material was available. A selection of representative quotes obtained during the home interviews is listed in Table S4 and referenced in the text by [Q#].

From the thematic analysis, three themes emerged from RQ‐1 and four themes emerged from RQ‐2 (Table 2).

**Table 2:** Research questions and related themes

Research question	Themes
RQ1. What are the current obstacles of child involvement in patient consultations in the context of the Child Brain Lab?	Different perspectives on obstacles to child involvement (parent, clinician, child)
RQ2. Which design features enable active child involvement?	Preparing for the consultation
	Transferring information
	Giving control and influence
	Providing and overview of their health

*RQ1: What are the current obstacles of child involvement?*

Three obstacles emerged that may explain children's passivity. Each stakeholder group had different views on these obstacles.

• Not feeling addressed on their level
*Patients* typically stated to trust their parents to act in their best interest during a consultation and that their parents would not keep information from them. One child mentioned that during the consultation, he would ‘zone out and wait for his turn’. This implied that he did not listen to what was being said because he assumed it was not meant for him. He also mentioned that he did like it when the doctor would talk to him. Other children gave similar statements. Observations showed that especially younger children's engagement level increased when they were directly addressed by the clinician.
  Children indicated that they did not always understand their doctor, though they typically also indicated that they did not necessarily mind [Q3,4].
*Parents* were generally satisfied with how the professionals involved them but were less positive about how their children were involved. According to parents, not all professionals gave comprehensible explanations that were directed at the child [Q5]. The parents' view was in line with the observations during patient consultations. Often, only a few general remarks were exchanged with the child.
  In contrast, *medical professionals* stated that it is often the parent who dominates the consultation. The professionals also report that they typically try to first involve the child, but that the child is not always in the mood to participate or contribute to the conversation [Q6].
  The professionals acknowledged that patients seemed more engaged when the doctor shared the test results directly with them, especially when the child could comprehend the message. Two medical professionals mentioned that children can be suspicious of clinicians or intimidated by the hospital environment and might therefore become somewhat shy or closed [Q7].
  Several medical professionals pointed out that young children or children with behavioural disabilities (e.g., autism) are typically not yet able to reflect on or to engage with abstract information [Q8]. This complicates engaging the child in the conversation, especially given the short time frame of the consultation.
• Needing energy and time to process information and experiences
  One *patient* was observed to ask many questions to the lab technician during his EEG test. However, after having to wait for the results, the child was no longer actively participating in the consultation. In other observations, patients lost their focus during the consultation.
*Parents* reported that hospital visits can take hours, depending on travel and waiting times; this limits the child's ability to participate [Q9]. Some *professionals* recognized this but explained that the hospital sometimes tries to combine tests and consultations on the same day to make it more manageable for the families.
  Some *parents* indicated that their child typically waits until after a test or a consultation to ask questions, likely because the child needed time to process the information. Similarly, the health play specialist stated that processing of the information and experiences of a full hospital day typically happens at home after the hospital visit.
• Not finding all information relevant to them
*Patients* seemed content with a basic and practical understanding of their condition and the reasons for going to the hospital (e.g., ‘because my head was too big’ or ‘to get better’). They were mainly concerned with the practical consequences of their condition and expressed less interest in why things were the way they were [Q10,11]. Two older patients mentioned that it would not change anything for them anyway.

The older child patients (>9 years) were especially concerned with how their condition affected their ability to participate in activities with peers and how they are seen by them [Q12]. Additionally, earlier experiences with the hospital also played a role in what they wanted to know. For example, one child specifically asked the doctor if she would have to have surgery again, mainly because the last time the anaesthetics made her vomit.

Two *medical professionals* (social worker and play specialist) emphasized that their main challenge is that children differ extensively in the level of understanding and communication preferences. They recognized some patterns: younger children prefer thinking about their own health in terms of their current symptoms rather than developments in the future. They are also more interested in the ‘what’ than in the ‘why’ because the latter is too abstract. A child and adolescent psychiatrist indicated that it is difficult to communicate with children about their mental health if they do not recognize their behavioural problem.

### 3.1 Additional findings on the importance of child involvement

In addition to identifying obstacles that complicate child engagement, the interviews and observations also provided insight into the different stakeholders' views on the importance of child involvement in general. Parents and medical professionals indicate that patient participation is important to them, whereas patients express more varied opinions.

*Parents* expressed that through active involvement, a child is better informed and prepared, and less anxious of treatments [Q1]. Some also mention that children will better adhere to instructions that come directly from a professional or that active participation in the consultation helps children in becoming more independent when growing up.

*Medical professionals* indicate that the importance of child involvement for them lies in knowing the perspective of the patients on experienced severity of symptoms and burden of treatment. They mentioned that they need this information, in addition to the clinical results, to advise on the most appropriate treatment plan for this specific patient. A plastic surgeon mentioned how, if a child would not see a specific deformity as a problem (yet), she might not find it necessary to operate on the child immediately.

However, in some specific cases, typically in the case of psychiatric disorders, clinicians find it counterproductive or even harmful to discuss everything with the child present, for example, when it is expected that children may not be able to understand explanations, when topics are too sensitive to be discussed in the presence of the child (i.e., bedwetting, behavioural problems) or when an emotional reaction from the parents is expected from which the child should be protected.

The *patient* observations revealed them taking on a passive role during the consultations. No questions were asked by them. Although they generally agreed with having to be present, they seemed to view the consultation as directed at parents and find it somewhat boring. Particularly younger children (<8 years) did not seem to pay attention to the conversation all the time. Data from the sensitizing booklets and observations confirm this (Table 3). One patient suggested that a short phone call would suffice; another asked if he could play cards during the consultation, such that he ‘had something to do’.

**Table 3:** Examples of answers to the question: ‘Does your child ask questions during the consultation?’

	‘Nathan’ (7)	‘Ruben’ (10)	‘Thomas’ (6)	‘Maike’ (12)	‘Marieke’ (6)	‘Simon’ (10)
Parents: Does your child ask a lot of questions about his or her condition or about clinical tests? (On a scale from 1 to 5; taken from sensitizing material when available)		2/5	2/5	3/5	2/5	2/5
	‘He asks 1000 questions a day’	‘He already knows a lot about his treatment’	‘Doesn't ask out of ignorance’	‘Asked when she would notice improvements for example’	‘Sometimes she asks a question, sometimes she doesn't’	‘We prepared him for what would happen’
Child patients: Do you ever ask a question to the doctor? (Taken from the sensitizing material when available)		‘Always’		‘Sometimes’	‘Sometimes’	‘Never’
		(However, this was not in line with the response during the interview)				
Observed questions: (Number of questions the patients asked during observed consultations)	0	No consultation observed	0	0	No consultation observed	0

Quotes are obtained from observations and interview data. Fictitious names are used.

Some *patients* indicated that they did not mind their limited involvement, which was confirmed by the parents [Q2].

*RQ2: Which design features enable active child involvement?*

Stakeholders' responses on the different design features (Figure 1) and the full concept design (Figure 2) can be divided into four subthemes:

- Preparing for the consultation
- Conveying information about the brain or (brain) disorder
- Giving control and influence
- Conveying individual test results

### 3.2 Preparing for the consultation

Both parents and medical professionals appreciated design features that allowed the children to reflect on the consultation beforehand as it helped children to form opinions or prepare questions they likely would not think of during the consultation. Additionally, they valued features where the preparation at home followed the same structure as the consultation. Also, all stakeholders were unanimously positive about a preparation feature where each stakeholder could visually mark (via a sticker) a topic (i.e., a brain function) that they would want to discuss during the consultation (Figure 2, feature 6) [Q13]. The parents positively appraised that they could prepare for the consultation and engage with medical information at home, together with the child.

Some medical professionals saw value in sharing (parts of) the test results before the consultation to allow families to prepare at home more effectively. It should allow families to process the information before discussing it. In the interviews, however, both parents and professionals expressed concerns about the risk of parents overthinking or misinterpreting the shared information, potentially leading to unnecessary stress [Q14]. To avoid this, such a report needs careful drafting and tailoring to the communication preferences of the individual parent. Medical professionals considered such a process as time‐consuming and thus difficult to realize. One clinician mentioned that even ‘quick to provide’ and ‘nonsensitive’ information, such as ‘what topic will be discussed’, would require a change in workflow with preparation of consultations longer in advance.

Patients indicated that they enjoyed seeing their own test results (particularly pictures of their brain), even when they did not fully understand them. Visual information seemed to catch the child's attention better than verbal information, thereby increasing engagement.

### 3.3 Conveying information about the brain or (brain) disorder

While discussing the design features, several professionals underscored the importance of finding the balance between providing too little or too much information to children. Both can lead to increased anxiety. They reported that finding this balance depends on a combination of the children's personality, previous hospital experiences and cognitive capacity [Q15].

One professional mentioned that children with autism tend to fixate on small details in explanations and to take abstract analogies too literal. For other children, such analogies (e.g., ‘the brain as the computer of the body’), can, however, be helpful tools for making messages more concrete and comprehensible.

In general, both parents and medical professionals stated that the parents know best how their child will react to certain information. Therefore, both appreciated design features that allowed patients to explore information at home. Parents and professionals saw potential in features that allow for dividing the information over time to better suit the attention span of young children. Some parents and professionals pointed out they liked how the information at home was consistent with what the child would face in the hospital. For instance, they valued that by collecting the brain puzzle pieces to complete the puzzle in the concept design, the child better understands that different tests are needed to gain a complete overview of their condition and level of functioning.

Some parents expressed a need to receive the information before their child receives it. Roughly half of the parents mentioned that they wanted to be able to hide or edit certain information, while the other half preferred total transparency towards their child. One professional and one parent explicitly warned against overprotective parents [Q16].

The parents were unanimous about wanting to receive more information than their children so that they could answer potential questions that the children might have. Also, they strongly preferred engaging with medical information together with their child, or at least with them present in the background, to support their child if needed. Consequently, the parents were positive about the design features that triggered child–parent interaction at home. For example, some parents preferred a design that contained (additional) tangible elements that could be explored together over designs that were purely digital. However, patients' interests were triggered more by digital and interactive elements than by traditional media. For example, they preferred that their avatar could be unlocked digitally when scanning the piece with a smart device, even if this would mean that their avatar would not appear on the puzzle pieces.

Some parents indicated that they appreciated that the children were not only informed about *what* would happen in the hospital but also about *why* certain procedures were needed. The older child patients expressed annoyance about not knowing *why* certain procedures were needed and that they would like to receive this information. Parents and medical professionals suggested that younger children possibly share this annoyance but were less articulate about it. Here, a one‐ or two‐sentence‐long explanation was said to suffice. It was appreciated that more information was available but hidden; information is only displayed when there the child shows an interest.

Patients mainly expressed an interest in practical information (especially younger children) or information that related directly to their daily life and their peers (especially older children). Both parents and child patients mentioned how they appreciated preparatory videos that would show exactly what would happen in the hospital [Q17]. One parent and child expressed how they especially liked videos where a peer tells the story.

Responses on the first design sketches also showed that apart from *what* and *why* information was being transferred, it also mattered *how* information was transferred. All respondents were positive about design elements that made it more fun for the patients to engage with the information. They responded enthusiastically towards the playfulness and interactivity triggered by the design features.

The patients' responses to the concept design indicated that they also valued the personalization and aesthetics of information. When asked why they liked the brain pieces (i.e., the medium of information about brain functions), most children mentioned that it featured themselves on it (i.e., their personal avatar) or that it looked nice in general [Q18,19]. Only one child made a remark related to the actual information (‘that I learn more about the brain’).

### 3.4 Giving control and influence

Several professionals and parents mentioned that child engagement is bolstered by the child having some control and input over the situation. For example, features where the child could choose a topic of discussion or could prepare a question in advance were well received by both groups. Giving ‘the child a stage’ to join the conversation in this way was seen as one of the most valuable ideas within the concept design [Q20].

When providing children forms of control, for example, through gamified elements, it was considered important to avoid triggering false expectations. One professional mentioned that, when asking the opinions of patients, it is important to avoid the impression that they could decide about treatments by themselves. Similarly, one parent warned against giving the patients more control in game elements than they have in reality.

More trivial forms of control (e.g., an avatar) were said to make a difference by several professionals [Q21]. Similarly, one 6‐year‐old patient mentioned that he liked the magnetic resonance imaging session because he could choose the colour of the ambient lighting.

### 3.5 Conveying individual test results

Medical professionals initially saw potential in simplifying test results into a visual gradation system. Such a system would give age‐appropriate information about the level of functioning of a patient, for example, by using traffic light colours, Emojis, stars or basic graphs. From the professionals' perspective, children understand such a gradation system and it provides an accessible overview of their level of functioning that they would otherwise lack. They saw the concept design as a tool that would help them and the patients to discuss their health in a broader perspective as opposed to focussing on a specific disorder [Q22].

During the second enriched interviews, the professionals indicated that such a system should be automatically generated based on numerical test scores, to avoid subjective interpretation and to reduce preparation time on their part. Some of them did point out that they wanted to be able to ‘tweak’ the overview to make sure that irrelevant results do not distract from the main message they wanted to convey.

Most parents were initially cautiously positive about a gradation system. After engaging with the star score stickers in the home interviews, however, some parents expressed concerns that a score overemphasizes the child's impairments, in particular, when such a score was auto‐generated and based on numerical data alone. Especially with young children, who tend to overestimate their own capabilities, ‘labelling results as bad or good’ could then be needlessly confronting [Q22]. A more useful metric for them would be ‘improvement’ compared to previous results.

Generally, both professionals and parents were cautious about showing ‘negative information’ to the child. A comparison with the general population average was said to be demotivating for those who score below it. Showing the child's results as ‘poor’ in any overview triggered questions on appropriateness and desirability. Children can associate such a gradation system with a school report. As one child pointed out: ‘I want to see my score because then I know what I can improve’. Although this implies that they understand the star score design, it also shows that they can interpret it in a way that a low score is their own doing and that it can be influenced by them.

Most clinicians eventually rejected the concept of a visual gradation system as they considered reducing the patient's condition to such a score without providing context as unfeasible for the professional and undesirable for the patient.

## 4 DISCUSSION

Here, we described the first steps to develop a tool that is specifically designed for 6–12‐year‐old children who visit the *Child Brain Lab*. Through this tool, we aim to prepare them for the lab tests and inform them about their results in a manner that is engaging and personally meaningful. The overall goal of the tool is to support shared decision‐making by increasing child involvement and active participation during consultations, while also supporting counselling by the medical professionals. We set out to identify (1) obstacles for patient involvement and (2) design features that enable patient participation.

In line with what was shown previously, both parents and medical professionals find it important to think about ways to increase patient involvement during clinical tests and consultation visits.^6^, ^22^, ^23^ Parents mentioned that proper preparation reduces anxiety, instructions might be taken more seriously by the patient if they are provided by the professionals instead of by themselves and that actively engaging children helps them to become independent and to take responsibility for their treatment process in the future. Physicians argue that they use the patient's perspective to make clinical decisions and therefore need to hear the children's experiences and thoughts. Several studies have shown that children want to be involved in their medical process.^9^ Interestingly, in the current study, patients, when asked, did not express a clear wish to take on an active role. However, during the observations, it became clear that when they were addressed directly, engagement increased, and they positively appraised this. Several reasons for their lack of involvement were identified, that is, patients are not addressed at their level of understanding, need energy and time to process the information and experiences before making an active contribution to the consultation and do not find all information so relevant to them. Parents and professionals each perceive different obstacles to involvement of the child during the consultation. Where parents at times felt that professionals did not actively involve the child in the conversation, clinicians may find that parents dominate the conversation. However, both groups recognize that there are situations where the involvement of the child may be counterproductive or harmful.

From our second research question, it emerged that several design features were suggested by different stakeholders to play an important role in enabling active child involvement. Both professionals and parents found it important to provide preparatory information on test results, but the information must be adapted to the children's personality, previous hospital experiences and cognitive capacity. Also, both groups argued that parents play an important role in deciding what information should or should not be shared with their child. For example, parents wanted to receive more information than their child, to be able to answer their child's questions. Design features that support this are those that allow patients to explore information on their own at their own pace and that also allow child–parent interaction (e.g., digital tools in combination with tangible elements). Moreover, a combination of age‐appropriate visit preparation (videos of hospital procedures and hospital room interiors) and information about how the brain functions was well received, thereby integrating *what* and *why* questions. Design features that gave a sense of control were highly valued by professionals and parents, for example, features that allow the child to choose a topic of discussion or to prepare a question in advance, but also more trivial choices during the test procedures, such as the colour of the lighting in a room. Importantly, these latter examples were also rated positively by the patients themselves and increased engagement. Other design features that made patients wanting to engage with the tool were learning about experiences from peers (other children feature in videos), personalization (their own avatar) and collection (the different brain pieces forming a brain puzzle).

Clinicians saw potential in design elements that enable translating test results on different domains into a visual gradation system for patients and their parents to evaluate at home. Features like traffic light colours, Emojis, stars or basic graphs seemed appropriate. When actually working with an automated rating system, professionals were no longer convinced that this is the way forward, as the nuance was lost, and they felt that information was given without them providing the necessary context on how to interpret the ratings. Also, several parents questioned the value of such rating overviews after they and their children engaged with the star score stickers in the home interviews. They expressed concerns that a simple star score overemphasized the child's impairments. In contrast, design features that allow children and their parents to form opinions, formulate questions and prepare for the consultation (e.g., place a sticker on different brain functions) were positively evaluated by parents and medical professionals. Moreover, it helped children when preparation at home followed the same structure or timeline as the testing and consultation.

To summarize, we aimed to go a step further than most previous attempts to involve paediatric populations in value‐based healthcare by actively focussing our attention to the triad of patient, parents and medical professionals and by applying tools with specific design features to stimulate child involvement.^1^, ^4^, ^7^, ^8^ To enable all three stakeholders in the triad, we aimed at designing a tool that offers a common language and a role for all stakeholders and relates the different parts of the patient journey. Such a tool is needed as parents as well as medical professionals may lack the insight and skills to enable and support the child to participate, and the capacity of the child influences the level at which he/she participates in care.^1^, ^11^, ^13^, ^24^

Currently, the narrow time frame of the consultation does not suit most patients' needs to understand, process and immediately respond to medical information and the implications.^6^ The tool that we tested showed that child patients' engagement is supported by design features that extend the time frame of interaction of the patient journey beyond the consultation (i.e., from preparing for the tests, getting first impressions of test results, preparing for the consultation, to processing the outcomes of the consultation). Design features that (1) help to transfer information interactively (e.g., a combination of tangible and digital elements, different levels of information, involve peers to share experiences) and (2) give control and influence during the consultation (e.g., place a question mark sticker on a piece of the brain puzzle at home) were highly valued by all stakeholders.

How to provide health information and share test results through a rating system appeared more complicated. Sharing (parts) of the test results in advance may help families to prepare for the consultation more effectively. However, concerns were expressed by professionals that it is difficult for parents and children to correctly interpret the information without context and by parents that a rating system may overemphasize impairments of their child and may be perceived as judgemental. This part of the tool needs further investigation. Important issues to consider when developing tools like the ‘*Self‐Portrait*’ are the (developmental) age of the child, the child's level of functioning on different domains and the role of the parents in controlling *what* information is given *when* and *how* to the patients.

Several limitations must be considered when interpreting our findings. First, patients were recruited from the Sophia Children's Hospital and most were in a follow‐up phase of care. Participants' views may be different during more acute phases of the child's illness, when care plans still must be established, or when they are suffering more from their condition. Second, the small sample size limits the generalizability of our findings. Third, the feedback that was collected on design ideas varied greatly. Although this may indicate a well‐balanced response group, it may also be a sign of undersaturation of the findings, especially as the target group is so varied in age, developmental stage and medical condition.

A wide range of conditions from different medical specialties were included in this study because we aimed at involving all multidisciplinary teams of the Pediatric Brain Center. Although these children have a wide variety of conditions, they share many physical and mental health problems. The current project does not address the specific conditions but focuses on the shared problems of these children. The results showed general insights on how to improve participation, but also more specific findings, for example, that physical problems may need a different approach than emotional or behavioural problems. Further research will have to explore these differences.

Finally, at times, we encountered a lack of interest or reluctance of children to articulate their views despite the use of enriched interviews. This may have caused some loss of data.

## 5 CONCLUSION

Several obstacles were identified that complicate child engagement that may differ between stakeholders. Promising design features were identified that have the potential to play an important role in enabling active child involvement. These findings show that applying principles of human‐centred design research and codesign, specifically focused on children with a variety of disorders that involve brain functioning, in a clinical setting can bring the triad of patients, their parents and medical professionals together around a tool that provides a shared language and focus, which are key prerequisites to increase child engagement.

## CONFLICT OF INTERESTS

The authors declare no conflict of interests.

## AUTHOR CONTRIBUTIONS

Paul Meulendijks: *Designed and conducted the research under supervision and drafted the manuscript*. Neeltje van Haren, Mathieu Gielen and Marie‐Lise van Veelen: *Initiated study idea, supervized research, drafted and revised the manuscript*.

## Supporting information

Supporting information.

Supporting information.

Supporting information.

Supporting information.

Supporting information.

Supporting information.

## Data Availability

The data that support the findings of this study are available from Marie‐Lise C. van Veelen‐Vincent (m.l.c.vanveelen@erasmusmc.nl) upon reasonable request.
