# Supplementary figures and images for: A self‐portrait: Design opportunities for a tool that supports children's involvement in brain‐related health care
Source: Health Expect. 2022 Jan 27;25(5):2235–45. doi: 10.1111/hex.13431 (PMC9615056; doi:10.1111/hex.13431)

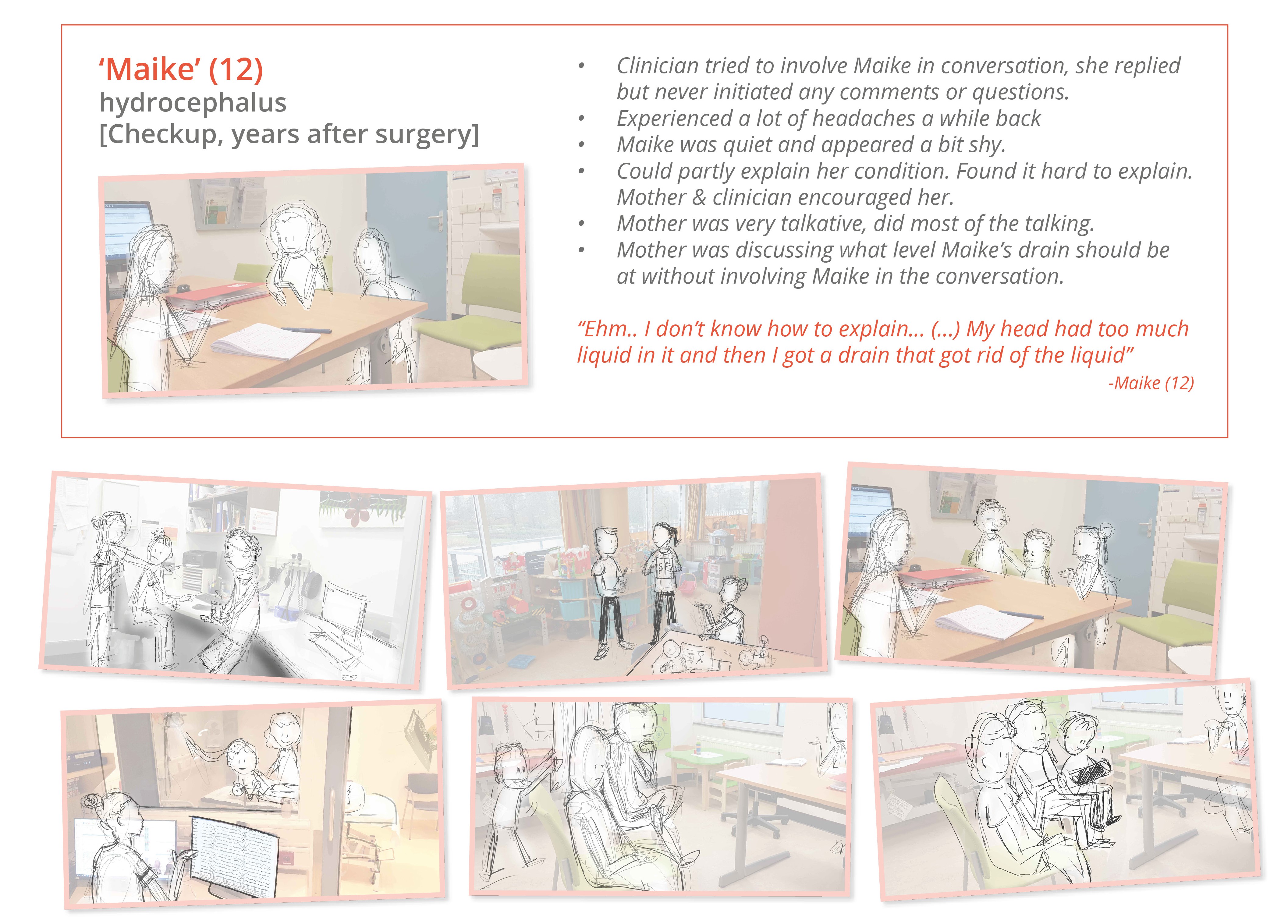

Supplement: Supplementary file 1 — Supporting information. [file HEX-25--s005.jpg]

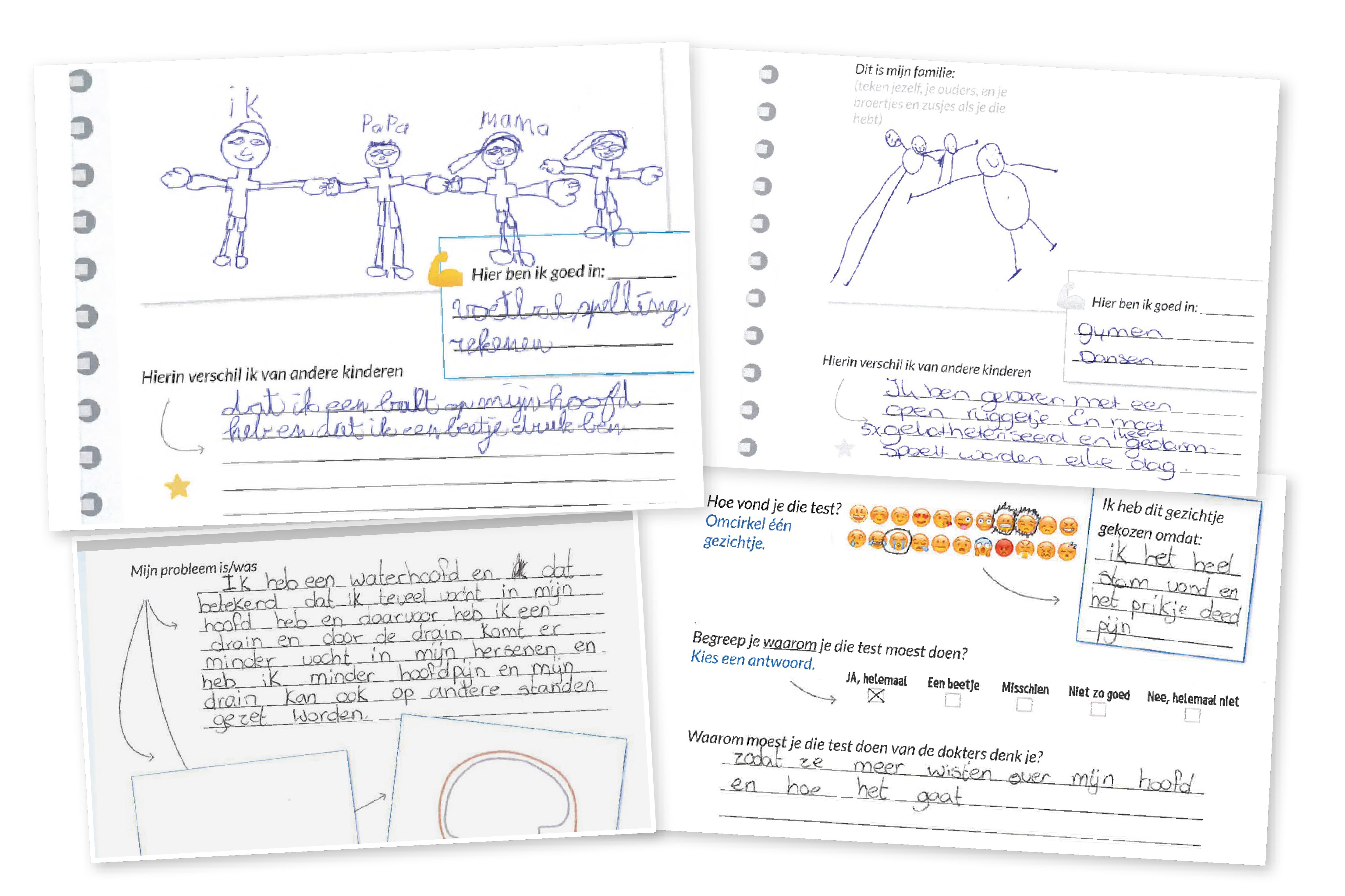

Supplement: Supplementary file 2 — Supporting information. [file HEX-25--s003.jpg]

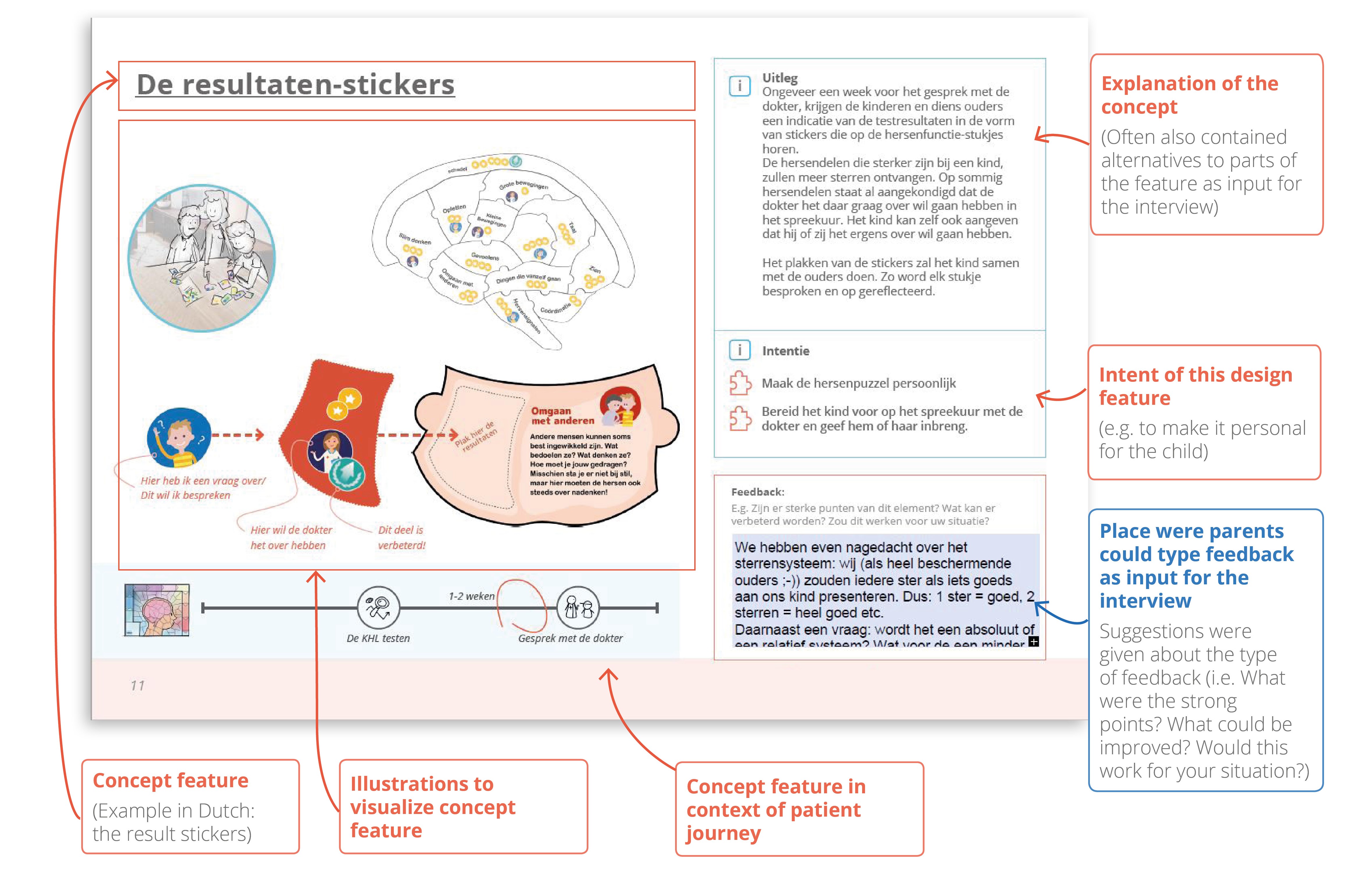

Supplement: Supplementary file 3 — Supporting information. [file HEX-25--s002.jpg]

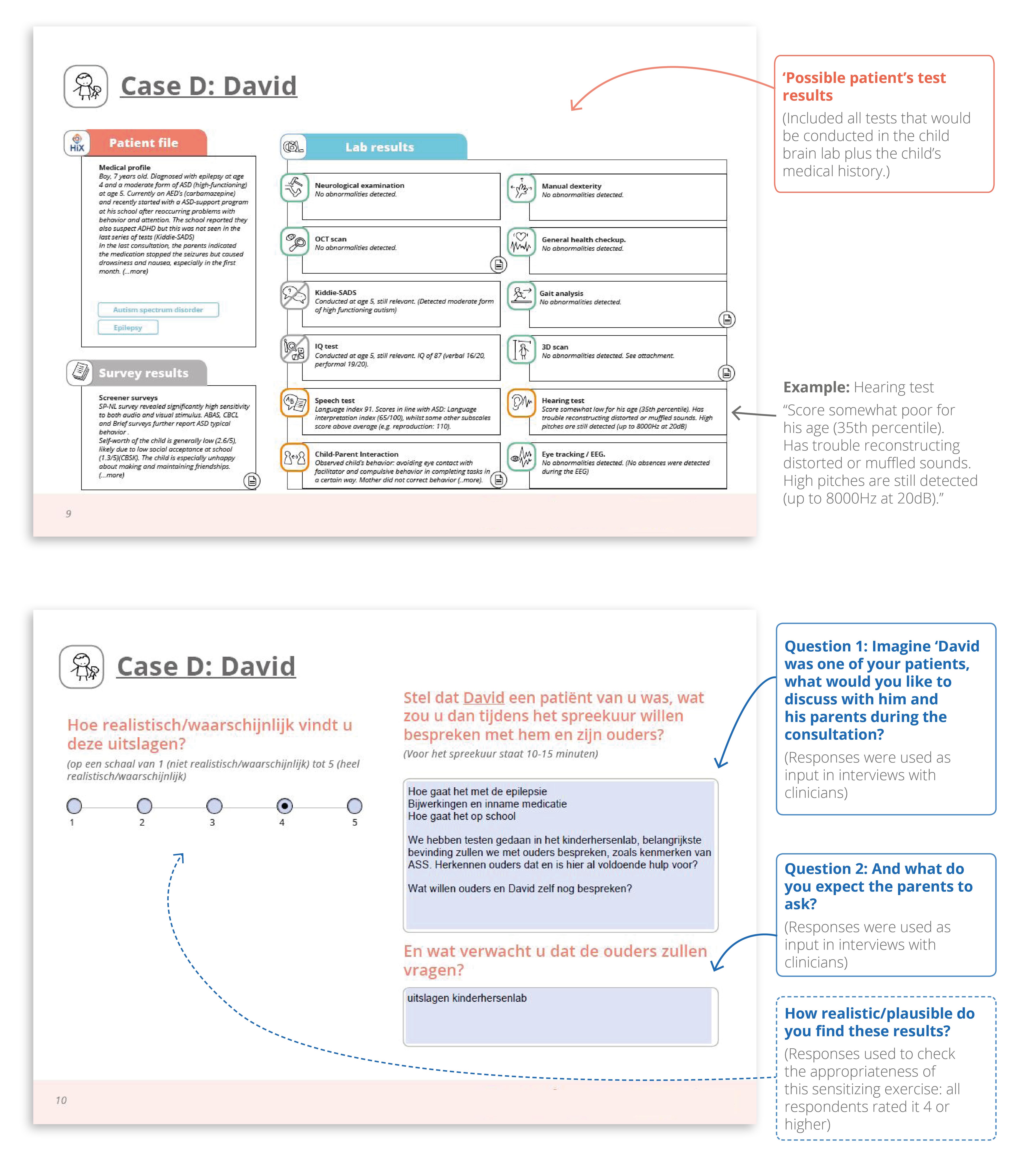

Supplement: Supplementary file 4 — Supporting information. [file HEX-25--s001.jpg]

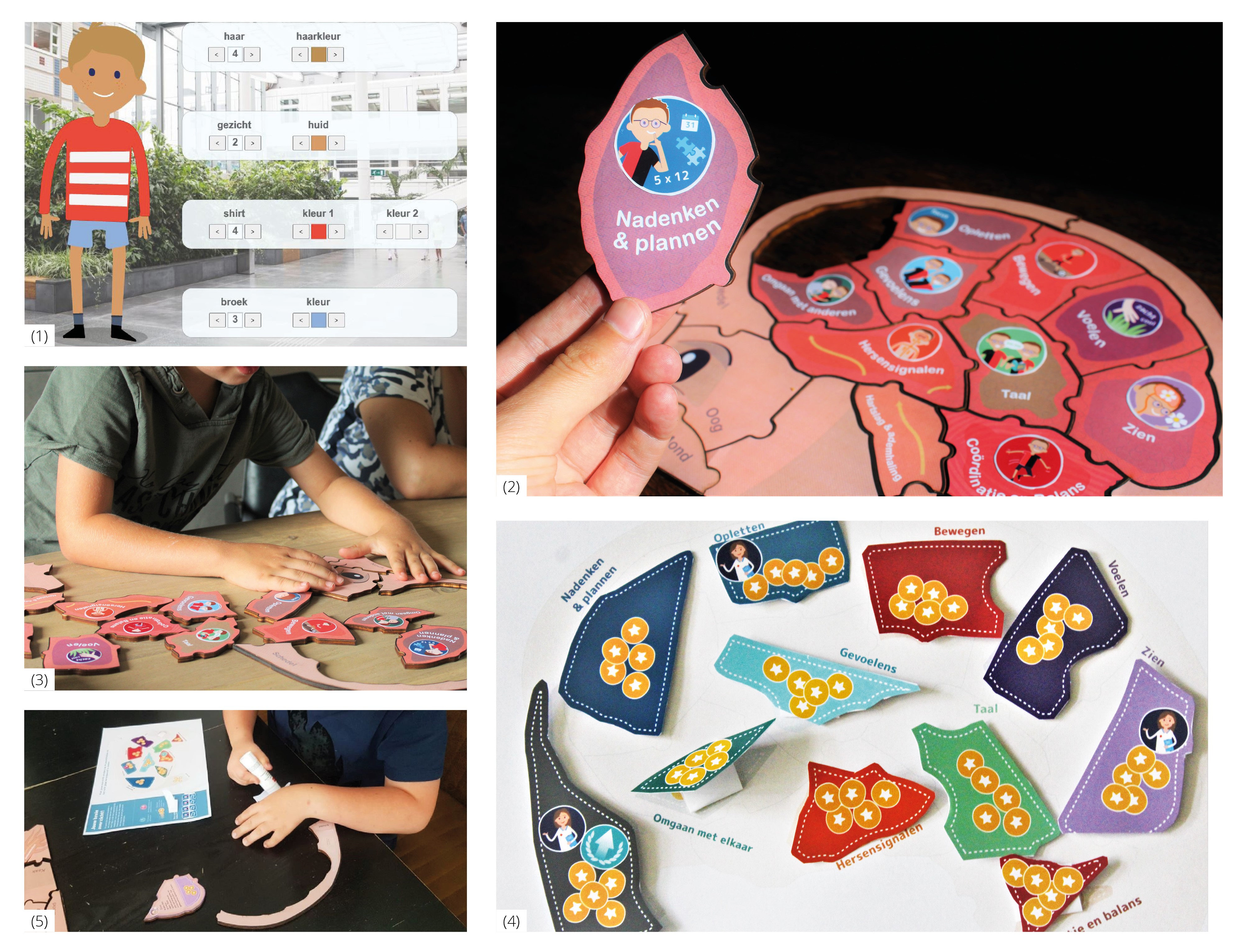

Supplement: Supplementary file 5 — Supporting information. [file HEX-25--s004.jpg]
