# Supplementary material for: A self‐portrait: Design opportunities for a tool that supports children's involvement in brain‐related health care
Source: Health Expect. 2022 Jan 27;25(5):2235–45. doi: 10.1111/hex.13431 (PMC9615056; doi:10.1111/hex.13431)
Supplement: Supplementary file 6 — Supporting information. [file HEX-25--s006.docx]

**Supplemental Material**

**Supplemental Figure legends**

**SFigure-1: An impression of the notes and sketches made during the contextual observations.** Shortly after each observation, the rough notes and sketches were elaborated upon digitally. Data from the observed consultation with ‘Maike’ is shown as an example. ‘Maike’ is a fictitious name.

**SFigure-2: An impression of a patient’s sensitizing booklet and their responses.**

**SFigure-3: An impression of feedback material for the 1st and 2nd enriched online interviews.** A slide deck explained the concept(s), divided in roughly 10 different features which followed the order of the patient journey. This is an impression of the Result Sticker feature. On each page, a participant could give their feedback prior to the interview. The structure of the slide deck was similar for the 1st and 2nd enriched online interview.

**SFigure-4: An example of a fictitious patient’s test results as sensitizing material for clinicians.** Four such ‘fictive results’ were created. Each medical professional received two of these, depending on their clinical expertise.

**SFigure-5: The physical prototype of the brain puzzle concept, which was used during the 2nd enriched home interviews.** A prototype was made and personalized for each child by using an avatar they created prior to the interview. 1) An avatar creator tool, which was developed to allow the child picking their personal avatar 2) A close-up of the brain puzzle with the functional domains featuring the child’s avatar. 3) A child playing with the puzzle during the home interview. 4) The ‘result’ stickers based on their parents’ or clinician’s perception of the child’s current functioning. 5) A child attaching the result stickers to the back of the puzzle pieces.

**Supplemental Tables**

**Stable-1:** *Demographic and clinical characteristics of patients and their parents and the time they invested in various research activities*

| **Patients (N=9) & their Parents (N=15**)  (Estimation in min) | *Contextual observation* | *Contextual interviews* | *Enriched interviews (online)* | *Enriched interviews (online)* | *Enriched interviews (home)* |
| --- | --- | --- | --- | --- | --- |
| ***Total*** | 225 | *350* | 180 | 300 | 360 |
| ***Nathan (m, 7),*** *Epilepsy, attends special care education.* | 130 (40) |  |  |  | 90 (45) |
| *Parents of Nathan* |  |  |  | 60 |  |
| ***Alex (m, 5),*** *Craniofacial disorder, occasional headaches* | 30 |  |  |  | 40 |
| *Parents of Alex* |  |  | 60 | 70 |  |
| ***Thomas (m, 6),*** *Craniofacial disorder, switching to special care education, minor speech impairment* | 15 | 50 (30) |  |  | 50 (30) |
| *Parents of Thomas* |  |  |  | 50 |  |
| ***Simon (m, 10),*** *Craniofacial disorder, No other signs or symptoms* | 15 | 70 | 50 |  | 90 |
| *Parents of Simon* |  |  |  | 50 |  |
| ***Ruben (m, 10),*** *Rare neuro-immune disorder, chronic fatigue* |  | 90 (40) |  |  | 90 |
| *Parents of Ruben* |  |  | 70 | 70 |  |
| ***Maike (f, 12),*** *Hydrocephalus, mild cognitive delay* | 10 | 60 |  |  |  |
| *Mother of Maike* |  |  |  |  |  |
| ***Marieke (f, 6),*** *Spina bifida & Hydrocephalus, occasionally needs wheelchair* |  | 20 (15) |  |  |  |
| *Mother of Marieke* |  |  |  |  |  |
| ***Pien (f, 4),*** *Craniosynostose* | 15 |  |  |  |  |
| *Mother of Pien* |  |  |  |  |  |
| ***Selçuk (m, 6)*** *Schizencephaly, ventriculoperitoneal shunt* | 10 |  |  |  |  |
| *Parents of Selçuk* |  |  |  |  |  |
| ***Sanne (f, 24)*** *Single-sided deafness, Cleft lip and palate (reflected on experiences during childhood)* |  | 60 |  |  |  |

Merged cells indicate both parents and child were interviewed or observed. In some cases, the child was only involved in the activity for a shorter duration of time, in which case that time is indicated between brackets. Names are fictitious.

**STable-2:** Background information on the background of the medical professionals and the time they invested in various research activities.

|  | **Medical professionals (N=15)**  *(Estimation in min)* | *A: Contextual observation* | *B: Contextual interviews* | *C: 1st enriched interviews* | *D: 2nd enriched interviews* |
| --- | --- | --- | --- | --- | --- |
|  | **Total** | 180 | 290 | 140 | 320 |
| *Researcher (N=2)* | senior researcher child psychiatry 1 |  | 50 |  |  |
|  | senior researcher child psychiatry 2 |  |  | 60 | 60 |
| *Clinicians (N=8)* | neurosurgeon | 90 |  |  | 50 |
|  | plastic surgeon 1 |  | 30 |  |  |
|  | plastic surgeon 2 |  |  |  | 60 |
|  | neurologist 1 | 30 |  |  | 30 |
|  | neurologist 2 |  |  |  | 60 |
|  | general paediatrician |  |  |  | 60 |
|  | child psychiatrist 1 |  | 60 | 60 |  |
|  | child psychiatrist 2 |  | 30 | 20 |  |
| *Supporting staff (N=5)* | Nurse practitioner |  |  |  | 10 |
|  | Social worker |  | 90 | written feedback |  |
|  | Health play specialist |  |  |  |  |
|  | Technologist (EEG) | 60 |  |  |  |
|  | Medical photographer |  | 30 |  |  |

Merged cells indicate that these medical professionals were interviewed during the same session.**STable-3A**: Question guide for analysing children’s responses to the physical prototype

| item | Question | Answer options* |
| --- | --- | --- |
| 1 | Did they seem excited when seeing the puzzle pieces for the first time? | Scale (1-4)** |
| 2 | Did they like the experience of puzzling? | Scale (1-4) |
| 3 | What did they like about making the puzzle? (If anything) | Text |
| 4 | Did they find the puzzle interesting? | Yes / No |
| 5 | What did they find interesting? (If anything) | Text |
| 6 | Did the puzzle trigger questions about the brain or *their* brain in particular? | Yes / No |
| 7 | Did they recognize their personal avatar on the puzzle, something they made / helped make? | Yes / No |
| 8 | Did they see their avatar as ‘themselves’ and not just an avatar? | Yes / No |
| 9 | Did they like their avatar being on the puzzle? | Scale (1-4) |
| 10 | Would they want to show the puzzle to their friends | Yes / No |
| 11 | What (if any) are the reasons for that? | Text |
| 12 | Did they generally seem to understand what the brain puzzle pieces were about and what they meant? | Yes / No |
| 13 | Could they at least partly understand the explanations at the back of the brain puzzle pieces when they read it or when it was read to them? | Yes / No |
| 14 | Did they intuitively understand that the brain puzzle pieces are all connected (and affect each other)? | Yes / No |
| 15 | Did they seem curious about learning more about the brain functions? | Scale (1-4) |
| 16 | Did they seem like they wanted to see their star score stickers when asked about it? | Scale (1-4) |
| 17 | How interested did they seem in their star scores after they have seen it? | Scale (1-4) |
| 18 | Did they want to put the star score stickers on their puzzle pieces? | Yes / No |
| 19 | Did the star score stickers trigger questions from the child about their own brain? | Yes / No |
| 20 | Did the child understand all the star scores and conversation stickers after a short explanation? | Yes / No |
| 21 | Was the child happy with their star score? | Scale (1-4) |
| 22 | Would the child want to receive another star score after future tests? | Yes / No |
| 23 | Did the conversation stickers encourage the child to ask questions to the doctor? | Yes / No |
| 24 | Would they want to show their star score to their friends? | Yes / No |
| 25 | In general, was the child more positive or negative about the stickers? | Positive / Negative |
| 26 | What (if any) were the reasons for that? | Text |
| 27 | Did they think the prototype (i.e. brain puzzle pieces + stickers) was something for children their age? | Yes / No |
| 28*** | Did they seem excited to be able to ‘scan their avatar to life’ feature? | Scale (1-4) |
| 29*** | Did they seem to prefer having ‘scannable silhouette avatars’ over having their own avatar on the brain puzzle? | Yes / No |
| 30*** | Did they seem excited to be able to watch videos through the piece scanning feature? | Scale (1-4) |

*Researchers could also fill in ‘I don’t know’ on all items.

**Four-point scale consisted of: Not at all (1), Not very (2), Somewhat (3), Very much (4)

***Items relate to an additional prototype where the children could ‘scan’ a puzzle piece about ‘seeing’ with silhouette avatar on it with a smartphone. On the screen, their personal avatar would then appear alongside with a video about perception and the brain from an educational children’s program.

**STable-3B**: Four examples of the questions guide for analysing children’s responses to the physical prototype

| **Item** | **Question** | **Answer researcher A** | **Comment researcher A** | **Answer researcher B** | **Comment researcher B** | **Answers Matched?** | **Agreement reached?** |
| --- | --- | --- | --- | --- | --- | --- | --- |
| 1 | Did they seem excited when seeing the puzzle pieces for the first time? | Somewhat | *The boy is curious and concentrated when puzzling (although a lot is happening with other siblings around).* | Somewhat | *Some curiosity and some reaction. His sisters also show quite some curiosity.* | yes | yes |
| 6 | Did the puzzle trigger questions about the brain or *their* brain in particular? | Yes | *When the researcher asks what is happening in their brains, he does not know what is meant. When his mom asks the same question, he is more interested and they talk about the signals that are sent to the brains. I think he is a bit shy.* | Yes | *Asked about the nervous system and the jaw ‘’what are those?’’. Though no further questions were asked.* | yes | yes |
| 10 | Would they want to show the puzzle to their friends? | Don’t know | *He says yes but his body language is very doubting / hesitant.* | Yes | *He mentioned he would.* | no | yes  Body language did not seem so contradicted when viewed again. |
| 22 | Would the child want to receive another star score after future tests? | Yes | *He likes to see the test results from another kid and wanted to see his own too.* | Don’t know | *This is not discussed.* | no | yes  It’s not discussed explicitly. It’s too big of a leap to make assumptions |

**STable-4:** A selection of representative quotes

| Q1 | ‘’The day of the surgery he was hopelessly crying on the couch: ‘What's’ going to happen, what will they do??’’  Parents of Thomas (6) |
| --- | --- |
| Q2 | ‘‘She is easy going in coming with us to the hospital. Sometimes she asks a question, sometimes she doesn’t. I don’t think she understands everything, but she seems to be okay with it so far.’’  Mother of Marieke (6) |
| Q3 | ‘‘I don’t really mind when my parents talk to the doctor. (...) I’ll zone out then and wait for my turn.”  Simon (10) |
| Q4 | ‘‘I prefer my mother to talk to the doctor because I don’t understand her myself.’’  Maike (12) |
| Q5 | ‘‘Currently we are not happy about how our child is involved. The conversations are about him but rarely with him.’’  Mother of Alex (5) |
| Q6 | ‘’A thing I don’t like is when parents try to take over the conversation (…) I always first talk to the child to see what they think and then to the parent. Not every child wants to say something, but you must give them the opportunity.’’  Neurologist 1 |
| Q7 | ‘‘It can also be that the child is intimidated. Of course, it can be a scary environment for a child. (...) They are afraid of the lab coats.’’  Plastic surgeon 1 |
| Q8 | ‘‘When I ask my patients ‘do you know why you’re here?’ The answer is almost always ‘No.’ (...) To understand their [behavioural] disorders, they need to be able to reflect on themselves; this is a skill children only start to develop around age 12.’’  Child psychiatrist 1 |
| Q9 | ‘‘It’s also that these consultations are scheduled at the end of the day. Then he doesn’t have the patience anymore to engage.’’  Parents of Thomas (6) |
| Q10 | [on whether or not wanting to know more about his condition] ‘‘(...) No thank you. I know what I have. I know I will have an infection again. I know I take medicine... I think I know enough.”  Ruben (10) |
| Q11 | Maike: ‘‘I did ask the doctor whether I had to have surgery again.’’  Mother: ‘‘she really hated that the aesthetics made her vomit after the surgery  last time. She hated that more than the surgery itself.’’  Maike (12) and her mother |
| Q12 | ‘‘I don’t want to present in front of the class about my condition. I don’t want them to  pity me. (...) They will ask more questions (...) The teacher will treat me differently  during P.E.’’  Maike (12) |
| Q13 | ‘’I would love it to make my consultations more playful in this way. It also forces me to do it together with the child: All pieces indicate what we are going to talk about at the start of the consultation.’’  Neurologist 2 |
| Q14 | ‘’I would not give more information. It quickly becomes like an ‘assessment report’ and even a few sentences can take on a life of their own inside the heads of our patients. It will be discussed during the consultation anyway.’’  Plastic surgeon 2 |
| Q15 | ‘‘The most challenging aspect of my job is that every child is different. (...) In a way, they are little jigsaws (…) What works for one child, might not work for another.’’  Health play specialist |
| Q16 | ‘‘You should protect parents from eh.. themselves. If you make information editable, parents might interfere with the clinical message from the doctor.”  Parents of Ruben (10) |
| Q17 | ‘‘We did not find the flyer so helpful, so we searched a bit and found a video from this other hospital. That helped a lot! (...) What was also nice is that another kid told the story’’  Parents of Alex (5) |
| Q18 | ‘‘I’d show it [the puzzle] to my friends because it looks nice and a lot of effort went into it. (…) I would say ‘Look, this is what the hospital has made!’’  Simon (11) |
| Q19 | Mother: ‘’Who is that on the piece?’’  Alex: ‘’Alex!’’  Mother: ’’That’s right, they put you on the puzzle!’’  Alex: ‘’Whoa! How did they do that?’’  Alex (5) and his mother |
| Q20 | ‘‘In this way you at least give him the feeling that he is involved and that we are doing this together. (...) You give him a stage (...) Well thought out!‘‘  Parents of Alex (6) |
| Q21 | ‘‘Having control, and being able to do something yourself is of great importance  in preparing children [through their patient journey].’’  Health play specialist |
| Q22 | I once had a 14-year-old patient that was telling me she was frustrated with failing some subjects (...) I dug up her MRI from when she was 3, showed it to her, and explained how her executive function ’the rational bit’ was affected. It helped her understand why she was great at literature but not at math. (...)  These puzzle pieces allow you to explain this at a much younger age. (...) Having that picture with the results [on the brain pieces], will be impactful. When you get angry at the situation, you can be angry at that specific piece, not at yourself.  General paediatrician |
| Q23 | ‘‘I found it difficult to go through this [the star score stickers] with Alex. He got 5 stars for ‘his skull’ but he still has headaches. On the other hand, he thinks he is great at moving around but not according to the score. I didn’t think it made sense to tell him otherwise now’’.  Mother of Alex (5) |
